# The Controlled Compound Layer of Ni-Coated Nitrided Pure Iron

**DOI:** 10.3390/nano11010031

**Published:** 2020-12-24

**Authors:** Qianqian Shen, Yu Zhang, Xuesha Li, Li Xiang, Chaoyin Nie

**Affiliations:** School of Materials and Energy, Southwest University, Chongqing 400715, China; qianqshen89@126.com (Q.S.); zhangy346@163.com (Y.Z.); lxs2019@email.swu.edu.cn (X.L.); lsguc_6303@163.com (L.X.)

**Keywords:** Ni-coated pretreatment, microstructure, compound layer, nitrided layer, effective hardened layer, wear resistance

## Abstract

In order not to sacrifice nitrided layer thickness and reduce brittle compound layer thickness, Ni-coated pretreatment was carried out with electrodeposition on a pure iron surface, followed by gas nitriding. The brittle compound layer thickness of duplex surface treated samples was reduced, and the nitrided layer thickness increased to 320 μm. The microhardness was 4 times harder, and the wear loss was reduced by 68% compared with the original material. The results indicate that Ni-coated pretreatment could effectively improve microhardness and wear resistance and realize the controlled microstructure of a brittle compound layer of pure iron without compromising nitrided layer thickness. Ni coating plays an important role in ammonia adsorption and decomposition, and in the transfer of active nitrogen atoms during nitriding.

## 1. Introduction

As a typical chemical heat treatment method, gas nitriding is widely used to strengthen the surface hardness, mechanical properties, and corrosion resistance of metal materials [1,2,3]. For nitrided pure iron, a compound layer of ε-Fe_2–3_N and γ′-Fe_4_N, and a diffusion layer is formed on the surface [4]. The ε-Fe_2–3_N phase is vulnerable to embrittlement and spalling during the use of nitride components, leading to accelerated damage and wear [5]. However, the existence of a diffusion layer increases the fatigue performance of nitriding parts [6]. Therefore, for the nitriding of steel, it is more desirable to obtain a thin compound layer (or no compound layer) and a thick diffusion layer.

A nanostructure surface on pure iron that was fabricated by surface mechanical attrition treatment (SMAT) could achieve 300 °C low-temperature nitriding [7], and the thickness of the SMAT pure iron nitrided layer was twice that of coarse-grained iron under the same conditions [8]. Pressurization technology also could increase the thickness of a nitrided layer, as reported by Wang et al. [9]. Chen et al. found that a rare-element (RE) catalyst of LaFeO_3_ perovskite oxide thin film could accelerate the nitriding rate of AISI 4140 steel at low temperature [10].

The cost of Ni is lower than precious metals and rare earth elements; and Ni is often used as an active component that is attached to ammonia decomposition catalysts, which contributes to improving ammonia decomposition efficiency [11,12,13]. Most of the research has focused on hydrogen energy as one of the clean energy sources [14,15]; only a few studies have focused on the relationship between nickel decomposition of ammonia and nitriding. For example, Inia et al. achieved low-temperature nitriding of pure iron below 325 °C by preparing a nanoscale nickel layer on a pure iron surface [16,17]. Van Voorthuysen et al. deposited a Ni layer with a thickness of 25 nm by electron beam evaporation on pure iron with a thickness of 250 nm and extended the Fe–N phase diagram boundary from 350 °C to 240 °C [18]. Based on a patent [19], Somers et al. conducted a series of studies to investigate the effect of nickel coating on the nitriding/carburizing hardening of stainless steel and its corrosion resistance. Brink and others of his team predeposited Ni on a stainless steel surface to study its magnetic properties [20]. Whereas these studies were based on nanometer-thick nickel coatings of low-temperature nitriding, the structure and properties of pure iron with slightly thicker nickel coatings are rarely studied.

Based on the above, this study fabricated an Ni coating thickness of several micrometers pretreatment that was coupled with gas nitriding technology on pure iron. The microstructure of the nitrided layer, the microhardness, and the wear resistance were investigated. Duplex surface treatment technology was expected to reduce the compound layer thickness on the surface of the pure iron while ensuring the nitrided layer thickness.

## 2. Materials and Methods

Bulk pure iron with a dimension of 50 mm × 20 mm × 5 mm was used. The iron was annealed at 900 °C for 1 h to eliminate the effect of mechanical deformation and obtain homogeneous coarse grains. The oxide scale on the annealed pure iron was then mechanically exfoliated with SiC sandpaper.

**Figure 1:**
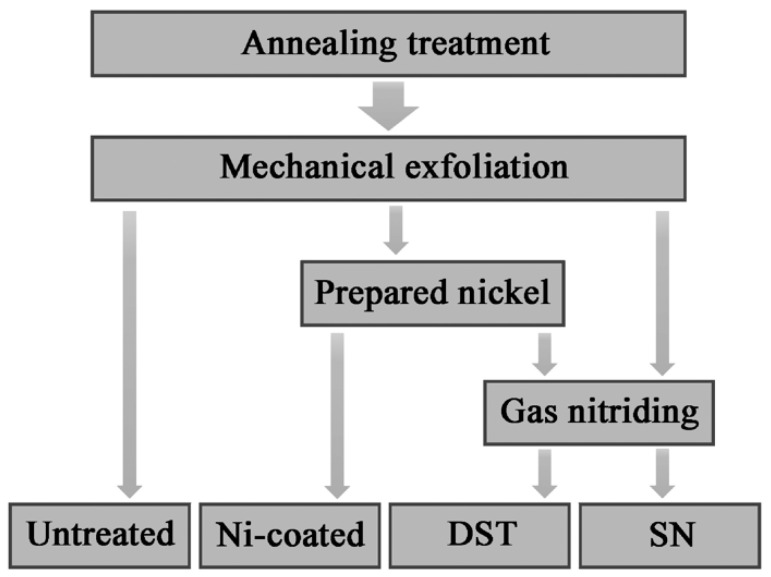
Flowchart of the sample fabrication processes.

Four types of samples were fabricated with different surface treatments, as shown in Figure 1. The nitrided samples involved two types: simplex nitrided (SN) and duplex surface treated (DST) samples. Combining pre-electrodeposited Ni coatings and gas nitriding, the duplex surface treatment was achieved. The Ni coatings were electrodeposited by direct current power with the following technological process: electrolytic degreasing, acid pickling and activating, nickel pre-plating, and nickel plating. The step of nickel pre-plating was performed in a solution containing 220 g/L nickel chloride hexahydrate (NiCl_2_·6H_2_O; Aladdin, AR) and 100 mL/L hydrochloric acid (HCl; Chuandong, AR) at a current density of 5 A/dm^2^ for 4 min. The nickel-plating step was performed in a solution containing 380 g/L nickel sulfamate (H_4_N_2_NiO_6_S_2_·4H_2_O; Macklin, 99%), 32.5 g/L nickel chloride hexahydrate (NiCl_2_·6H_2_O; Aladdin, AR), and 32.5 g/L boric acid (H_3_BO_3_; Sinopharm, AR) [21] at a current density of 1 A/dm^2^ for 1, 5, 10, 15, and 20 min, respectively. The water used in the experiments was up to the standard of I level. Taking nickel plating time as a variable, the weight of nickel coating covered per unit area of pure iron was 5.76 × 10^−3^, 13.27 × 10^−3^, 26.55 × 10^−3^, 34.70 × 10^−3^, and 54.03 × 10^−3^ mg/mm^2^, respectively, and corresponded with DST samples numbered as DST-1, -5, -10, -15, and -20, respectively.

Gas nitriding treatment was conducted in a tailored horizontal tubular sintering furnace with a 180 sccm flowing high-purity NH_3_ atmosphere at 500 °C and ordinary pressure for 5 h, subsequently cooled down in the nitriding chamber under an N_2_ protection atmosphere. According to the principle that ammonia is soluble in water, but nitrogen and hydrogen are not, the ammonia decomposition rate of the SN sample and some of the DST samples was measured by an ammonia decomposer in the nitriding process.

An HVS-1000A Vickers microhardness tester (Huayin Test Instrument Co. Ltd, Laizhou, China) was used to measure microhardness along the cross-sections with a 25 g load and on the surface with a 25–500 g load and a holding duration of 10 s. Each surface microhardness value was equal to the average of the 5 microhardness measurements, and the error was calculated.

Reciprocating wear tests were conducted on a CSM ball-on-disc tribometer. The tests were carried out under dry sliding wear, with a 150 m sliding distance, a 6 cm/s sliding speed, and a 10 mm wear scar length. A fixed load of 10 N was used with a GCr15 steel ball (6 mm in diameter) as a counterpart. Before and after the wear tests, the samples and counterparts were ultrasonically cleaned with absolute ethyl alcohol and dried. The mass of each sample before and after the test was weighed by a 1/10,000 precision analytical balance, and the mass loss was calculated as the wear mass.

The cross-sections of all samples were chemically etched with a 3% alcohol nitrate solution and analyzed with a JSM-6510LV scanning electron microscope (SEM; JEOL, Tokyo, Japan) equipped with an energy dispersive spectrometer (EDS). The surface of the Ni-coated samples was characterized by a JSM-7800F thermal field emission scanning electron microscope (FESEM; JEOL, Tokyo, Japan) equipped with an EDS. The wear scars of the samples were also observed by the SEM.

To accurately obtain phase information of the compound layer, the top surface layer containing the Ni element was removed by mechanical grinding. Phase analysis was performed on a XRD–7000 X-ray diffractometer (XRD; Shimadzu, Tokyo, Japan) by using Cu-Kα radiation (λ = 1.54 Å) operating at 40 kV and 30 mA.

## 3. Results and Discussion

### 3.1. Microscopic Morphology

**Figure 2:**
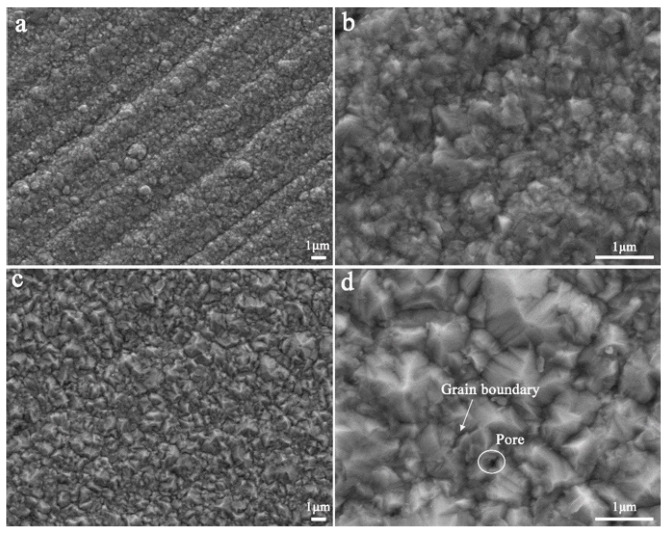
The samples with 5.76 × 10^−3^ mg/mm^2^ Ni coating (**a**,**b**) and with 34.70 × 10^−3^ mg/mm^2^ Ni coating (**c**,**d**).

Figure 2 shows the surface morphology of the Ni-coated pure iron. Due to the thin Ni coating, the scratches that occurred during mechanical exfoliation to remove the oxide scale of the annealed pure iron surface could be observed (Figure 2a). An agglomeration phenomenon occurred on the surface of the pure iron with the 5.76 × 10^−3^ mg/mm^2^ Ni coating, about 1 μm (Figure 2a), but most of the area of the Ni coating surface was nanoparticles of relatively uniform size (Figure 2b). With the increase of Ni coating thickness, no scratches similar to those in Figure 2a are observed in Figure 2c. The Ni coating on the surface of the pure iron with the 34.70 × 10^−3^ mg/mm^2^ Ni coating was uniformly distributed, about 500 nm–1 μm, which was larger than that of the coating of 5.76 × 10^−3^ mg/mm^2^ Ni due to nucleation growth (Figure 2d).

**Figure 3:**
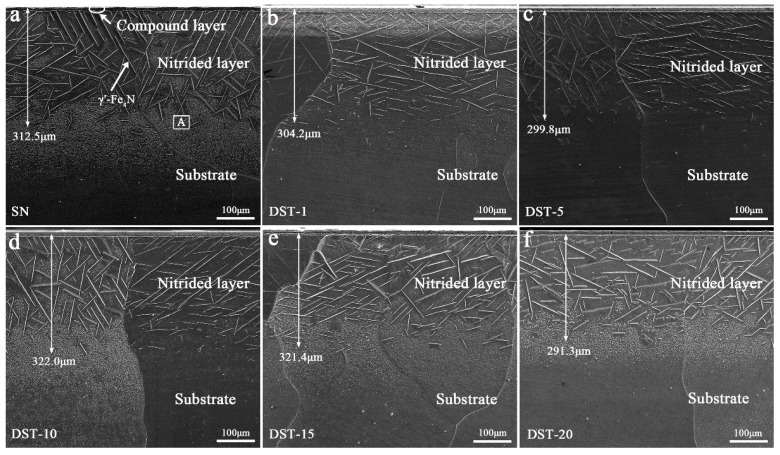
SEM images of cross-sections of the simplex nitrided (SN) sample (**a**) and duplex surface treated (DST) samples (**b**–**f**).

**Figure 4:**
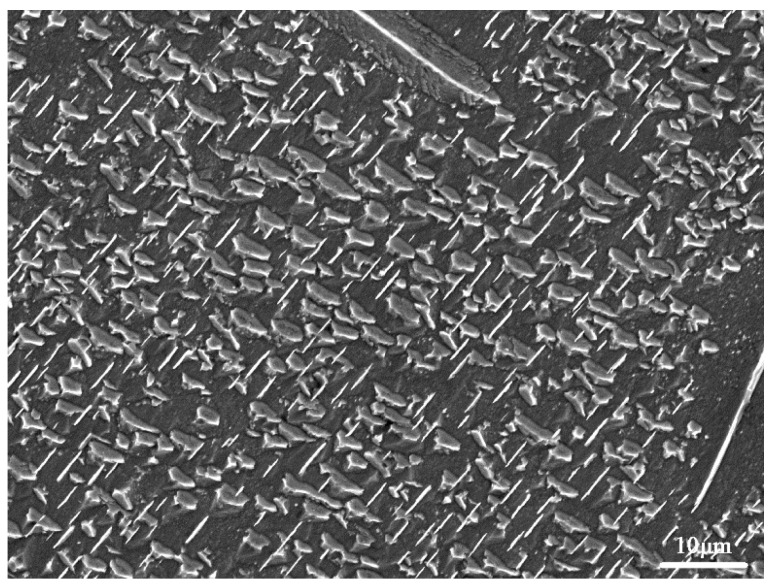
High magnification morphology of the box A area in Figure 3a.

After nitriding, the microstructure of the nitrided layers on the SN sample and DST samples was characterized and is shown in Figure 3. For all the nitrided samples, the nitrided layer included the compound layer and the diffusion layer. The needle-like nitrides (γ′-Fe_4_N) precipitated in the diffusion layer of the DST samples were shorter than those in the SN sample but had a similar γ′-Fe_4_N layer thickness of about 300 ± 10 μm. For the DST-10 and DST-15 samples, the nitrided layer thickness was up to 320 μm. According to the previous literature of [22,23,24], the short needle-like morphology was α″-Fe_16_N_2_ nitrides, also precipitated on all of the nitrided samples (see the high magnification image in Figure 4).

**Figure 5:**
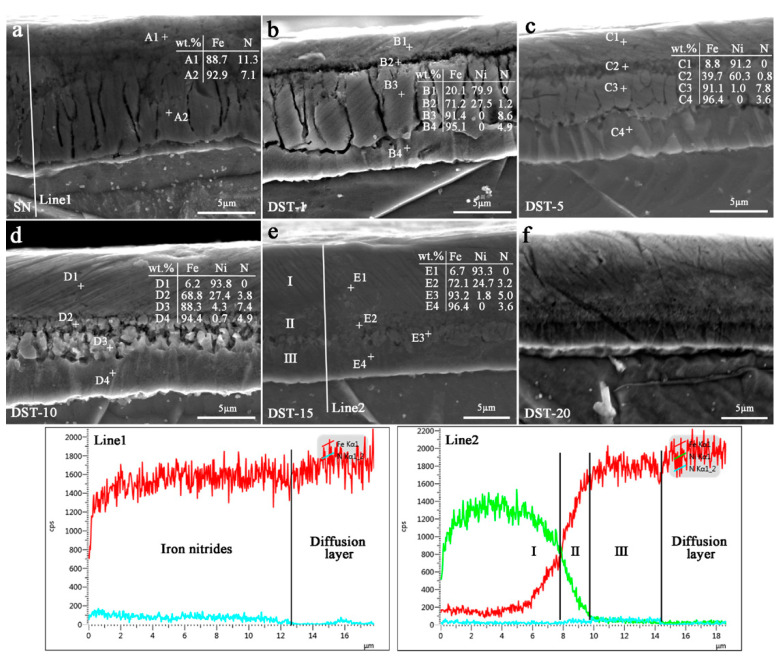
High magnification morphology, EDS element points, and lines of the surface layer on the SN sample (**a**) and DST samples (**b**–**f**).

The only difference is that the surface compound layer was decreased with the Ni coating thickness increase in Figure 5. According to the equilibrium phase diagram of Fe–N, the EDS data of elements and line1 showed that the SN sample surface was a compound layer consisting mainly of ε-Fe_2–3_N (Figure 5a). For the DST samples, the interface between the Ni coating and the pure iron substrate existed in an Ni-rich and Fe-rich region, where coupling occurred to form (Fe, Ni)_x_N during the nitriding process [25]. Thus, the EDS data of points and line2 showed that the surface layer of the DST samples was more complex than that of the SN sample (as shown in Figure 5b–f) and was mainly divided into three categories: (I) the top layer of Fe–Ni, (II) the secondary layer of iron nickel nitrides ((Fe, Ni)_x_N), and (Ⅲ) the iron nitrides layer near the diffusion layer. The Ⅲ layer also was divided into a porous compound layer and a transition layer (Figure 5b–e).

The surface of a nitrided alloy is the place with the highest nitrogen concentration [26]. In the nitriding process, an Ni coating not only protects pure iron from being oxidized by the trace oxygen in the atmosphere, but also serves as a catalyst to decompose absorbed ammonia, thus promoting nitriding efficiency [18]. However, it can be seen from Figure 5 that the N concentration in the surface layer of the pure iron substrate decreased with the increase in Ni coating thickness. This shows that the thickness of the Ni coating was too thick, which would hinder the diffusion of the active nitrogen atoms produced by ammonia decomposition to the pure iron substrate. The diffusion coefficient of N atoms in the ε nitrides was smaller than that in α-Fe [27]. Therefore, although a Ni coating of several micrometers thickness could prevent the diffusion of a large amount of N to the iron substrate, the N provided by it still satisfies the nitride’s growth kinetics. Furthermore, the diffusion of N in the pure iron substrate is mainly attributed to its ferrite grain boundary, rather than the Ni concentration gradually decreasing in the Fe-rich region [25]. This may be the reason that all nitrided samples are close in their needle-like diffusion layer thickness in Figure 3.

Ammonia in a nitriding environment is mainly divided into direct thermal decomposition ammonia and ammonia adsorbed on the surface of nitriding parts, and the thermal decomposition of the latter provides the active N atoms involved in nitriding [28,29]. In previous studies, Yin et al. pointed out that catalytic decomposition of ammonia by Ni was better than Fe [30]. Compared with a pure iron surface, an Ni coating composed of nanoparticles could increase the specific surface area of the entire sample and the amount of ammonia adsorbed on the sample surface. The grain boundaries between Ni nanoparticles and the pores generated during nucleation provide diffusion channels for N atoms. However, with the increase of the covered Ni content, i.e., the increase in Ni coating thickness and the grain size of Ni particles, the specific surface area would decrease, which may reduce the ammonia adsorption capacity compared with the Ni coating with the high specific surface area.

**Figure 6:**
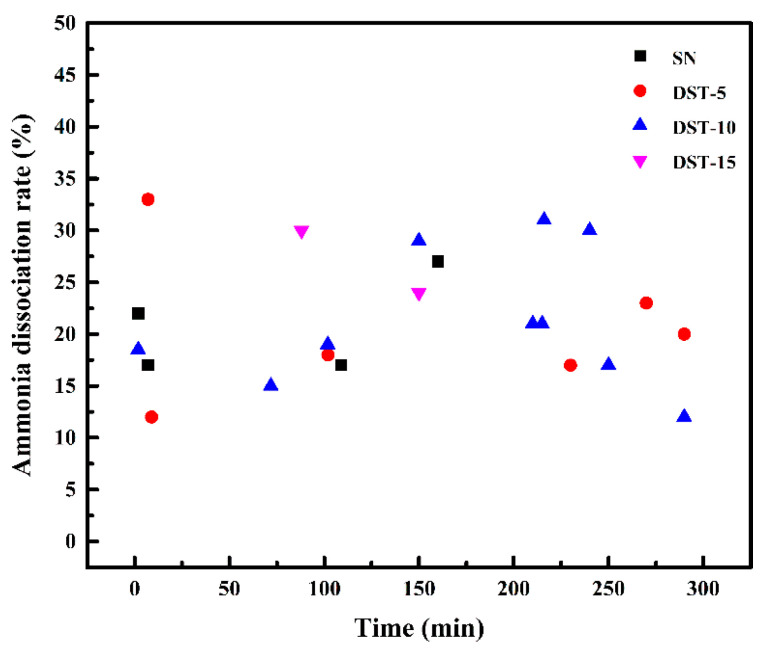
The variation in ammonia dissociation rate of the SN sample and DST samples as a function of nitriding time.

Nitriding potential is one of the factors that influence the growth kinetics of nitriding; it is influenced by pressure and is inversely proportional to the ammonia decomposition rate [31]. Increasing pressure could have achieved increased nitriding potential [32]. As this work was carried out under ordinary pressure, the influence of pressure on nitriding potential was not considered, but the ammonia decomposition rate and the results are shown in Figure 6. The ammonia decomposition rate of all the samples fluctuated in a similar range. The existence of Ni coating seems to have had little effect on the ammonia decomposition rate, which may be limited by the ammonia flow, the complex atmosphere in the reaction chamber, and less content of the Ni coating in the small sample. The nitrogen production from ammonia decomposition was related to the flow rate of ammonia and tended to be constant as a large ammonia flow, which was determined by the reaction kinetics on the workpiece surface [31]. In any case, this confirms that it is not feasible to discuss previously described results in terms of the nitrogen potential in this study. This also proves that the key to improving the nitriding efficiency of Ni coating rests with the adsorption and diffusion stages of active N atoms.

### 3.2. Phase Analysis

**Figure 7:**
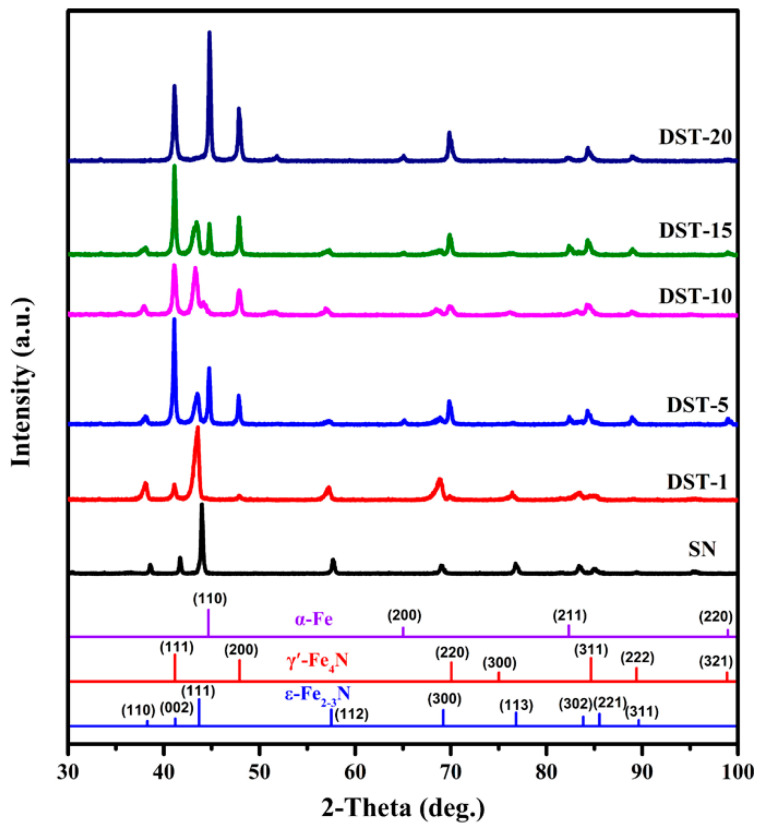
The XRD patterns of the SN sample and DST samples, excluding the Ni element layer.

Figure 7 shows the XRD patterns of the SN sample and DST samples, excluding the Ni element layer. The surface phase of the SN sample was mainly ε-Fe_2–3_N. However, the surface phase of the DST samples was relatively complex. For the DST-1 sample, the surface layer was composed of ε-Fe_2–3_N and a small amount of γ′-Fe_4_N. The surface layers of the DST 5–15 samples were composed of ε-Fe_2–3_N, γ′-Fe_4_N, and α-Fe, and the peak intensity of ε-Fe_2–3_N decreased gradually with the increase of Ni coating thickness, while the peak intensity of γ′-Fe_4_N and α-Fe increased gradually. The diffraction peak of the ε-Fe_2–3_N on the surface layer of the DST-20 sample disappeared, the phase consisted of γ′-Fe_4_N and α-Fe only, and the intensity of the α-Fe diffraction peak was stronger than that of other nitrided samples. Because X-rays have a certain penetration depth, it can be judged from the patterns that the iron nitrides compound layer of the DST samples with a thicker Ni coating was thinner, which is consistent with the morphological analysis in Figure 5. This indicates that the Ni-coated pretreatment had a regulatory effect on the structure of the surface compound layer.

Table 1 shows the lattice spacing of ε(111) and γ′(111) planes in the surface layer of DST samples, excluding the Ni element layer. As contrasting values—retrieved from JCPDS No. 49-1663 and JCPDS No. 06-0627—the lattice spacing (d in Table 1) of ε(111) was 2.0701 nm and that of γ′(111) was 2.1910 nm, respectively. It can be seen that all the lattice spacing of ε(111) and γ′(111) planes in the surface nitrided layers (d′ in Table 1) of the DST samples has been improved, and that the lattice spacing increased at first and then decreased with the increase in Ni thickness, which was similar to the variation trend of the transition layer thickness and γ′-Fe_4_N layer thickness seen in Figure 3 and Figure 5. The expansion of the lattice is related to the amount of N atoms diffused into ferrite. The lattice expansion on the ε(111) and γ′(111) planes was the most obvious on the DST-10 sample, indicating that the phase volume content of ε-Fe_2–3_N was the highest, which is consistent with the XRD pattern in Figure 7. It was also confirmed that the thickness of the Ni coating affects the N content of the compound layer and then the thickness of the diffusion layer.

### 3.3. Microhardness

**Figure 8:**
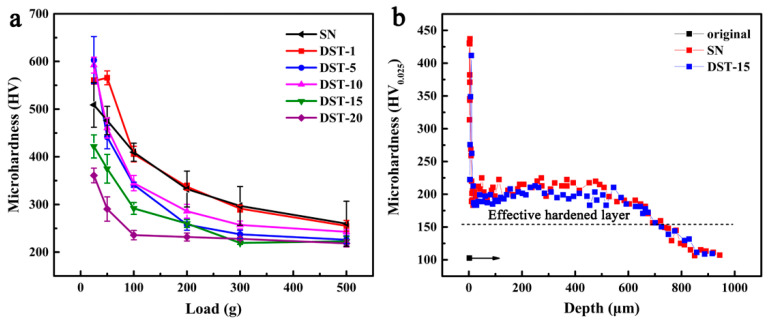
(**a**) Surface microhardness of the SN sample and DST samples; (**b**) Microhardness depth curves of the original material, the SN sample, and the DST-15 sample.

Figure 8a shows the surface microhardness of the SN and DST samples under a load of 25–500 g. The surface microhardness of all the samples decreased with the increase of load. The variation trend of the SN sample was similar to the DST-1 sample, and higher than the others under the given load from 50 g to 200 g. For DST 1–10 samples, the surface microhardness decreased slowly with the increase of load. Additionally, that of the DST samples decreased significantly with the increase of the Ni coating thickness under loads of less than 200 g, which was mainly caused by the decreased ε-Fe_2–3_N phase content on the surface. The microhardness of all samples was nearly similar and within the error range under loads of more than 300 g, which was mainly due to the microhardness of the diffusion layer.

As the porous compound layer on the surface of the DST-15 sample was extremely thin or even disappeared, and the nitrided layer was significantly thicker than that of the SN sample, the DST-15 sample was selected as the comparison sample. The microhardness distributions along with the depth of the SN sample and DST-15 sample are shown in Figure 8b. The hardness of the sample depends on the hardness of the phase [33]. It is well known that the hardness of ε-Fe_2–3_N is higher than γ′-Fe_4_N [33]. Therefore, the surface microhardness of the SN sample (~500 HV_0.025_) and DST-15 sample (~420 HV_0.025_) was about 5 and 4 times that of the original material (~103 HV_0.025_), respectively. The distance from the surface to a position 50 HV higher than the substrate hardness is defined as the effective hardened layer [10]. Due to the similar microhardness of the diffusion layers, the two types of nitrided samples had similar effective hardened layers.

### 3.4. Wear Resistance

**Figure 9:**
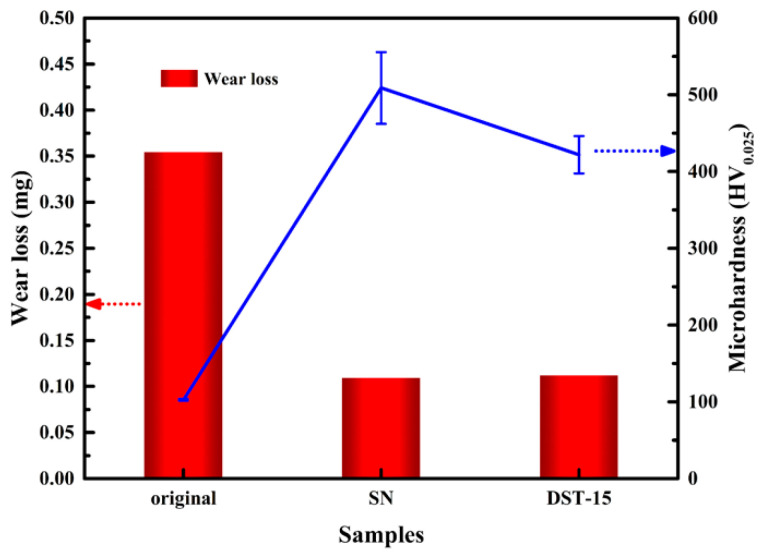
Comparison of the wear loss and microhardness of the original material, the SN sample, and the DST-15 sample.

The wear loss and microhardness of the original material, the SN sample, and the DST-15 sample are shown in Figure 9. It also can be seen that the microhardness of the SN and DST-15 sample was higher than that of the original material. Under the same conditions, the wear loss of the samples was inversely proportional to the microhardness and follows the Archard wear equation [34]:

W = KsP/P_m_, (1)

where W is the wear volume loss, s the sliding distance, P the load, P_m_ the microhardness of the sample, and K the friction coefficient. The wear loss of the SN sample and DST-15 sample had fallen by about 69% and 68%, respectively. Therefore, the wear resistance of the DST sample as well as the SN sample was obviously better than that of the pure iron sample, which had a positive effect on actual use-value.

**Figure 10:**
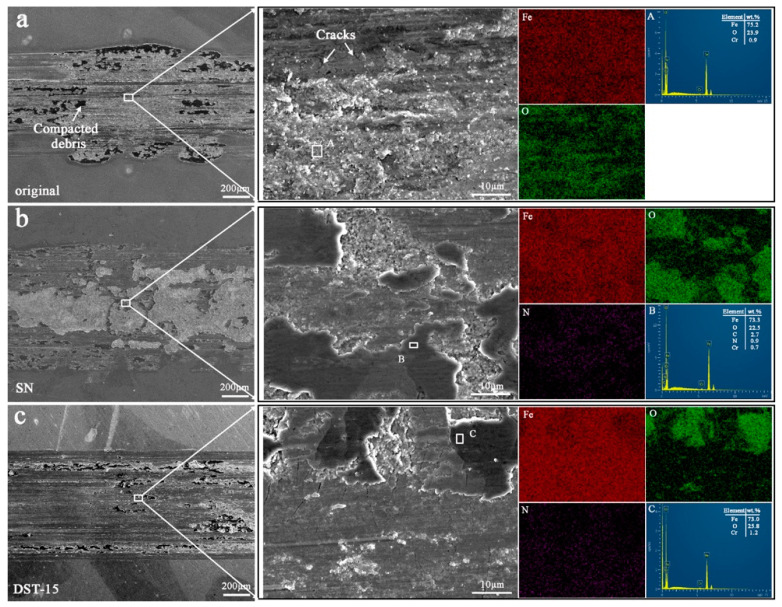
The low magnification morphology, high magnification morphology, and EDS element analysis of the wear scar of the original material (**a**), the SN sample (**b**), and the DST-15 sample (**c**).

Figure 10 shows the SEM morphology and EDS element analysis of wear scars of the original, SN, and DST-15 samples. From the SEM morphology of the original material, it can be seen that there were obvious furrows and cracks along a direction parallel to the sliding, and there were obvious piles of compacted debris on both sides and in the middle of the wear scar (Figure 10a). Only large areas of nitride spalling were observed on the SN sample (Figure 10b). Similarly, furrows and compacted debris were observed on the wear scar of the DST-15 sample, but these furrows were shallower than those of the original material (Figure 10c). The EDS mapping of the high magnification morphology of all the samples showed a large amount of O, indicating that oxidation occurred during the wear tests, especially where the black compacted debris was located, which confirmed that the compacted debris in the SEM images was oxides. A small amount of Cr element was detected in the point scanning, mainly from counterparts of the GCr15 steel ball, indicating that element transfer occurred during the wear tests. A large amount of N presented on the high magnification morphology of the SN and DST-15 samples, indicating that the wear scars of the nitrided samples remained in the nitrided layer and had not reached the substrate. The wear mechanism of the original material was mainly abrasive wear accompanied by adhesive wear and fatigue wear; the SN sample was mainly adhesive wear; and the DST-15 sample was mainly adhesive wear accompanied by abrasive wear.

## 4. Conclusions

The nitrided layer thickness of duplex surface treated (DST) samples was thicker than that of the simplex nitrided (SN) sample by up to 320 μm. In addition, the thickness and phase of the compound layer were achieved through a controlled change in the nickel coating thickness. The hardness and wear resistance of pure iron was improved by duplex surface treatment, which showed a positive effect on actual use-value. The pretreatment of Ni coating had the effect of adsorption, decomposition of ammonia, and transfer of active nitrogen atoms during nitriding, promoting the nitriding efficiency.

## Figures and Tables

**Table 1:** Lattice spacing was calculated from ε(111) and γ′(111) planes in the surface layer of the DST samples, excluding the Ni element layer.

Samples		DST-1	DST-5	DST-10	DST-15	DST-20
ε(111)	d′ (nm)	2.0760	2.0787	2.0878	2.0787	--
	(d′ − d)/d (%)	0.2850	0.4154	0.8550	0.4154	--
γ′(111)	d′ (nm)	2.1934	2.1944	2.1954	2.1933	2.1933
	(d′ − d)/d (%)	0.1095	0.1552	0.2008	0.1050	0.1050

## Data Availability

Data available on request due to restrictions eg privacy or ethical. The data presented in this study are available on request from the corresponding author. The data are not publicly available due to the data also forms part of an ongoing study.
